# Congenital Toxoplasmosis: The State of the Art

**DOI:** 10.3389/fped.2022.894573

**Published:** 2022-07-06

**Authors:** Lina Bollani, Cinzia Auriti, Cristian Achille, Francesca Garofoli, Domenico Umberto De Rose, Valeria Meroni, Guglielmo Salvatori, Chryssoula Tzialla

**Affiliations:** ^1^Neonatal Intensive Care Unit, Fondazione IRCCS Policlinico San Matteo, Pavia, Italy; ^2^Neonatal Intensive Care Unit, Medical and Surgical Department of Fetus – Newborn – Infant, “Bambino Gesù” Children’s Hospital, IRCCS, Rome, Italy; ^3^Department of Molecular Medicine, University of Pavia, Pavia, Italy

**Keywords:** congenital infections, *Toxoplasma gondii*, chorioretinitis, diagnosis, follow-up, pregnancy, neonate

## Abstract

Infection with the protozoan parasite *Toxoplasma gondii* occurs worldwide and usually causes no symptoms. However, a primary infection of pregnant women, may infect the fetus by transplacental transmission. The risk of mother-to-child transmission depends on week of pregnancy at the time of maternal infection: it is low in the first trimester, may reach 90% in the last days of pregnancy. Inversely, however, fetal disease is more severe when infection occurs early in pregnancy than later. Systematic serologic testing in pregnant women who have no antibodies at the beginning of pregnancy, can accurately reveal active maternal infection. Therefore, the risk of fetal infection should be assessed and preventive treatment with spiramycin must be introduced as soon as possible to reduce the risk of mother-to-child transmission, and the severity of fetal infection. When maternal infection is confirmed, prenatal diagnosis with Polymerase Chain Reaction (PCR) on amniotic fluid is recommended. If fetal infection is certain, the maternal treatment is changed to a combination of pyrimethamine-sulfonamide and folinic acid. Congenitally infected newborns are usually asymptomatic at birth, but at risk for tardive sequelae, such as blindness. When congenital infection is evident, disease include retinochoroiditis, cerebral calcifications, hydrocephalus, neurocognitive impairment. The diagnosis of congenital infection must be confirmed at birth and management, specific therapy, and follow-up with multidisciplinary counseling, must be guaranteed.

## Introduction

Toxoplasmosis is a systemic and cosmopolitan disease affecting about one third of the world population. The causative agent is *Toxoplasma gondii*, an obligate intracellular protozoan parasite, whose replication occurs in the intestine of cats and other felines, the only definitive hosts. Warm-blooded animals and humans are intermediate hosts.

Different strains have been identified, three main designated as type I, II, and III and other atypical, which differ in virulence and epidemiological pattern of occurrence. In Europe, 95% of human infecting *T.gondii* are type II, whereas in North America type II represents 43.9%, type III accounted for 18.2% and atypical strains accounted for the rest (1, 2). A recent study on genetic analyses of atypical strains revealed that a fourth clonal lineage (type 12 lineage) it is the dominant strain in wildlife of North America and accounts for the 46.7% of the isolated strains (3). The parasite genotype may play a role in determining the severity of disease: in South America the strains show greater genetic variability and are usually much more virulent (4, 5).

**FIGURE 1:**
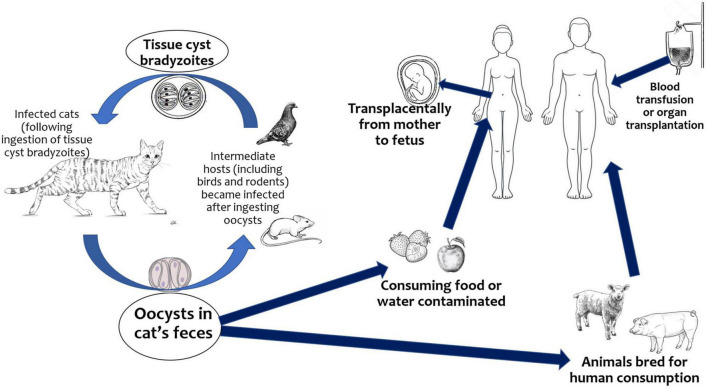
Life cycle of *Toxoplasma gondii.*

The infection is acquired mainly through the ingestion of raw or undercooked meat containing still viable cysts, through the ingestion of water, fruit, vegetables, shellfish, or by contact with earth contaminated by oocysts excreted in the feces of infected cats (Figure 1). *T. gondii* can also be transmitted *via* blood or leukocytes from immunocompetent and immunocompromised donors. The parasite persists lifelong as cysts in intermediate host (6).

With few exceptions, the acute phase in the immunocompetent adult is usually a subclinical or benign disease. In a minority there may be malaise, low-grade fever, and lymphadenopathy and chorioretinitis.

Primary infection induces the production of specific antibodies and lifelong immunity, but toxoplasmosis can reactivate in immunocompromised individuals (e.g., AIDS or treatment with corticosteroids).

In humans, the seroprevalence of *T. gondii* antibodies increases with age and varies considerably according to geographical location, health education, hygiene, food habits and climatic conditions, it decreased in the last decade due to a greater intake of frozen meat, better hygiene, progressive urbanization (7).

In Europe the IgG seroprevalence ranges from 30 to 50%, and in United States is about 9.1% of women of childbearing age. In South America, the prevalence varies from 30 to 80%, reaching 100% in the most advanced age groups of the poorest populations (8, 9). The overall global prevalence of acute infection in pregnant women is 1.1% but is higher in Eastern-Mediterranean region than in European region (7). The global IgM e IgG seroprevalence in pregnant women is 1.9 and 32.9%, respectively, with statistically significant differences between WHO regions (10).

Congenital toxoplasmosis occurs when maternal infection is acquired for the first time in pregnancy. During the phase of parasitemia, *T. gondii* may cross the placenta and enters the fetal circulation with a risk of fetal infection that increases with gestational age: this results in congenital infection affecting 25–30% of women treated during pregnancy (11). At 6, 18- and 30-weeks’ gestation of pregnancy, the risk of fetal infection is 2.2, 23, and 56%, respectively (12).

Incidence of acute infection among *T. gondii* seronegative pregnant women varies by geographic location; it is estimated in Austria, Sweden, France, and United States to be 0.8, 0.5, 2.1, and 0.2 per 1,000, respectively (8, 13, 14). The global incidence rate of congenital infection is estimated to be 1.5 cases/1,000 live births with higher burdens in South America, in some Middle Eastern countries and low-income countries and lower burdens in European countries (15). In particular incidence of congenital toxoplasmosis is 1.0/10,000 in Austria, 0.5/10,000 in United States and 2.9/10,000 live births in France (8, 13, 14).

The likelihood of fetal infection is low in the early pregnancy but increases in later stages. The chance of transmission increases by 12% per week of maternal gestation starting at 13 weeks of gestation (16, 17). The protective role of the placenta is more effective in the first trimester, allowing to the passage of parasites in less than 10% of cases. With the increasing of the vascularity, the placental barrier becomes more and more permeable, leading to parasite transmission in around 30% of cases in the second trimester, in 60–70% of cases in the third trimester, even more in the last weeks of gestation (18).

The mechanisms of *T.gondii* vertical transmission remain unclear, in particular the role of placenta-derived cytokines or chemokines. The syncytiotrophoblast has distinct resistance to T. *gondii* infection, at the level of attachment and post-entry replication, while cytotrophoblasts and extravillous trophoblasts seem not displaying the same kind of resistance, suggesting cell-type specific differences in mechanisms of resistance. In addition, trophoblasts respond to T. *gondii* infection through the specific induction of various cytokines and chemokines, including the robust induction of the regulatory T cell chemokine (19, 20).

The severity of fetal infection decreases with the increase of gestational age. During the first and second trimester, the infection may lead to miscarriage (around 3% of all cases) or still birth. Infected infants frequently show severe symptoms of congenital toxoplasmosis, with neurological disorders and ocular lesions, while in the latest phase of pregnancy the neonatal disease may be less severe or asymptomatic.

The higher risk of early and long term (within 3 years) clinical signs occurs in women who seroconverted between 24 and 30 weeks of gestation (about 10%); for infections occurring in the second and third trimester, the minimum risk is not less than 5% (12, 21). Therefore, dating maternal infection during pregnancy is of great relevance to establish the extent of fetal risks.

In this narrative literature review we provide an updated overview on diagnosis, therapy, and follow-up of toxoplasmosis in pregnancy and neonatal age.

## Methods

We searched PubMed^1^ for cohort, cross-sectional and case-control studies, reviews, expert consensus as well as case series or case reports published as articles or letters to the editor describing neonates with congenital toxoplasmosis. An extensive literature search has been performed up to 6 March 2022. The following keywords “Congenital Toxoplasmosis” AND “neonate” OR “infant” were searched as entry terms. We excluded all retrieved articles written in non-English language. Additional studies were identified by authors based on their knowledge on the field, if not already included by literature search.

## Neonatal Clinical Manifestations

In neonatal age, congenital toxoplasmosis is asymptomatic in 85% of cases. Infected newborn appears normal at the clinical examination, but he is at risk of developing ocular lesions later in life. In such a situation, it is hard to make a diagnosis without information on maternal serologic profile.

The classic triad described by Wolf in 1939 (hydrocephalus, intracranial calcifications, and chorioretinitis) is observed very rarely in present times. Neonatal manifestations, if present, may include hydrocephalus, microcephaly, intracranial calcifications, chorioretinitis, cataracts, convulsions, nystagmus, jaundice, petechiae, anemia, enlarged liver, and spleen, prematurity and severe intrauterine growth restriction with abnormally low birth weight (14, 21, 22) (Table 1). However, none of these symptoms is pathognomonic for toxoplasmosis and may suggest other congenital infections (CMV, Herpes simplex, rubella, syphilis) (6).

**TABLE 1:** Clinical features reported to be associated with congenital toxoplasmosis (14, 21, 22).

Systemic signs	Preterm birth*, small for gestational age*, rash* (petechial, blueberry muffin), sepsis like illness*, hepato/splenomegaly*, myocarditis*, hepatitis*, hepatic calcifications* jaundice, temperature instability, pneumonitis, lymphadenopathy
Laboratory abnormalities	Anemia*, thrombocytopenia*, CSF abnormalities like pleocytosis, elevated protein, eosinophilia, hypoglycorrhachia* increased level of liver enzymes or bilirubin level
Neurological signs	Macro or microcephaly*, hydrocephalus*, hypotonia*, palsies*, seizures*, psychomotor retardation*, spasticity*, SNHL*, intracranial calcifications*
Ocular signs	Amblyopia*, cataract*, chorioretinitis*, nystagmus*, optic nerve atrophy*, strabismus*, retinal scarring*, visual impairment*, microphthalmia, microcornea

*CSF, cerebrospinal fluid; SNHL, sensorineural hearing loss. Symptoms considered to be more common are indicated by an asterisk.*

The prevalence and severity of principal signs of disease are significantly different in the United States, France, other Western European countries, Israel, and South America (23–30). The reported rate of severe congenital toxoplasmosis is higher in the United States and South America in comparison to European countries due to different and more virulent *T. gondii* strains implicated and in the absence of antepartum treatment in the United States (1, 14, 31–34). In Israel the proportion of severe disease is higher than in Europe probably because of the lack of systematic prenatal screening and treatment but is lower in comparison to United States (30).

A systematic review of cohort studies by SYROCOT (Systematic Review on Congenital Toxoplasmosis study group) shows that, during the first year of life, 19% of infected infants developed at least one of two clinical manifestations: 14% had ocular lesions and 9% had intracranial lesions or both (26).

Among ocular manifestations, the most frequent is chorioretinitis and the retinal lesions are usually in the posterior pole (22, 35). Most frequently occurs after a reactivation of the infection and in cases where the macula is involved there may be a loss of visual function (22, 36, 37). Worsening of central vision, because of macula involvement, may recover after resolution of the inflammation (22). Chorioretinitis is commonly recurrent and relapsing, but these episodes are rarely associated with systemic signs or symptoms (22). For any additional week of gestation, in case of maternal primary infection, the risk of chorioretinitis decreased by 3% but increases by 2.1 times when maternal primary infection occurred before 20 weeks of gestation and by 3.6 times in infants with additional clinical manifestations at birth (38).

Other ocular disorders, reported apart from recurrent focal chorioretinitis, that can contribute to visual impairment are strabismus, microphthalmia, cataract, retinal detachment, optic nerve atrophy, iridocyclitis, nystagmus, and glaucoma (22, 36). Some of these manifestations develop as a consequence of retinal lesions or can be related to neurological involvement like hydrocephalus (36). Indeed, is reported that infants with severe ocular manifestations also present with severe cerebral damage (35).

The ocular manifestations in congenital infected infants in Brazil are more severe than in the United States and Europe (24, 35, 39). Brazilian infants developed chorioretinitis more frequently and the lesions are multiple, larger, and more likely located in the posterior pole than the European infants (24). Several studies suggest that the marked difference in prevalence and severity of ocular involvement in Brazil is due to different prevention protocols and infection with atypical *T. gondii* strains more virulent which are predominate in Brazil but are rarely found in other countries (24, 40–42).

Involvement of the central nervous system is demonstrated by calcifications that follow the phenomena of vasculitis and necrosis and affect mainly the periaqueductal and periventricular regions. Sometimes the hydrocephalus may be the only manifestation of congenital toxoplasmosis; observational data show a frequency of 31% when the mother did not receive any therapy, compared to 0.8% in the newborns of treated women (16). In the SYROCORT study the risk of intracranial manifestations was higher in Brazilian and Colombian infants in comparison to other countries (26).

## Diagnosis of Toxoplasmosis in Pregnancy

In pregnant women, when present, symptoms of toxoplasmosis are mild and non-specific (asthenia, low-grade fever, myalgia, and usually laterocervical lymphadenopathy), therefore, the diagnosis relies only on serological tests; in immunocompetent subjects, IgG, IgM, IgA, and IgE antibodies can be detected just after two weeks from the infection.

There is no consensus about serological screening for *T. gondii* IgG and IgM antibodies in pregnant women (5). It is rare in United States (8, 14), not currently recommended in Canada (43) but mandatory in France and Austria at the first trimester prenatal visit (13, 44, 45). The prenatal screening allows to identify anti-*toxoplasma* IgG seronegative women at risk of acquiring the infection in which the primary prevention is mandatory (5, 13, 44, 45).

Serum conversion is the most accurate marker of maternal infection (6). Positive IgG and negative IgM antibodies in the first and second trimester suggest that the infection has been acquired before the current pregnancy. When the first analysis is done during the third trimester, a negative IgM result cannot rule out an infection acquired in early pregnancy, since IgM could have become undetectable in a short time. Negative IgM rules out infection in the first two trimesters, but positive IgM is not always a certain marker of recent infection because IgM can persist beyond to years or can be non-specific IgM (6, 46, 47) (Table 2).

**TABLE 2:** Results of serological tests during pregnancy and their interpretation.

Scenario	IgG antibodies	IgM antibodies	Interpretation	Comment
1	Negative	Negative	Absence of immunity	Monthly serologic follow-up and 1 month after delivery
2	Positive	Negative	Infection acquired before pregnancy	- Repeat tests after 1 month to confirm previous infection - Stop follow-up if only IgG antibodies are positive
3	Negative	Positive	Initial seroconversion or IgM falsely positive	- Repeat test weekly - Second level tests - Eventual prenatal diagnosis - Neonatal follow-up
4	Positive	Positive	Acute infection or Persistence of IgM	- Date infection - Second level tests - Eventual prenatal diagnosis - Neonatal follow-up

Reference laboratories use second level tests: IgG avidity test, and immunoblotting test essential tools to help to date the infection (6, 48, 49). A high IgG avidity index suggests that the infection was contracted at least 16 weeks before; a low or intermediate avidity index is considered markers of recent/acute infection (50, 51). Furthermore, avidity grows more slowly if pregnant woman is subjected to a specific therapy, since treatment could delay IgG appearance and reduce avidity maturation (48).

The immunoblotting test for IgG and IgM could be used to evaluate IgM specificity and allows an earlier identification of specific IgG (6).

The monthly screening during pregnancy, until delivery, allows a timely diagnosis of the maternal infection, so as to start an early specific treatment, in an attempt to prevent transmission, or to reduce the risk of serious injury.

### Prenatal Microbiology Diagnosis

PCR techniques for detection of *T. gondii* DNA in amniotic fluid has revolutionized prenatal diagnosis of congenital toxoplasmosis. First, it makes possible an early diagnosis since PCR has a specificity and positive predictive value of 100%, furthermore, it avoids more invasive procedures on the fetus (17, 47).

However, amniocentesis should be performed only after the 18th week of pregnancy, four weeks after the estimated date of infection in a pregnant woman (17). A negative result does not fully exclude the presence of congenital toxoplasmosis, since negative predictive value is 98,1%; the rare false-negative antenatal diagnoses could be due to delayed transplacental transmission of parasites after amniocentesis or to very low parasite densities in amniotic fluid (52, 53). The risk of procedure-related fetal loss (or preterm delivery in more advanced gestation) is estimated to be less than 0.1% (54).

Early diagnosis of maternal infection plays a key role in clinical counseling, in assessing fetal risk and treatment options, and in planning prenatal diagnosis.

### Treatment of *Toxoplasma gondii* Infection During Pregnancy

**FIGURE 2:**
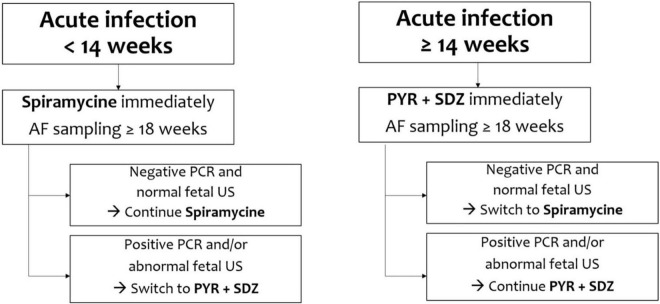
Pharmacological prevention of mother-to-child transmission.

There are many discrepancies in studies published between 1999 and 2006 on the efficacy of prenatal treatment in reducing the incidence and severity of congenital toxoplasmosis (55, 56). Since 2007, observational studies have evidenced a greater efficacy of the therapy when it is undertaken as soon as possible (ideally within 3 weeks from seroconversion) in order to prevent the transmission of the parasite to the fetus and reduce the risk and severity of fetal infection (Figure 2) (13, 26, 57–59).

In primary maternal *Toxoplasma* infection, acquired during the first 18 weeks of gestation, it is recommended the treatment with spiramycin, a macrolide that reaches significant placental concentration, and can reduce the frequency of vertical transmission, but is not effective for the treatment of fetal infection (60). A first randomized clinical trial describes a lower placental transmission rate by the association of pyrimethamine and sulfadiazine with folinic acid versus spiramycin, but enrollment was discontinued because of low enrollment and lack of additional funding (61). The combination of spyramicine and trimethoprim-sulfamethoxazole can cross the placenta and kill parasites in fetal tissues, therefore it seems to be more effective in reducing the risk of maternal-fetal transmission of *T. gondii* than spyramicine alone (62). When PCR is positive due to an infection acquired after the 18 weeks of gestation, the current gold standard is the association of pyrimethamine, sulfonamides and folinic acid (Table 3). This treatment cannot be used before 14 weeks of gestation for the potential risks of teratogenicity (17).

**TABLE 3:** Pyrimethamine–sulfonamides combinations for mothers.

Anti-*Toxoplasma* drug	Regimen use
Pyrimethamine	1 Tablet of 50 mg daily
Sulfadiazine	3 Tablets of 500 mg twice daily
Folinic acid	2 Capsules of 25 mg per week
**OR**	
Sulfadoxine–pyrimethamine combination	Capsules equivalent to Fansidar (500 mg/25 mg) must be prepared: 2 capsules per week
Folinic acid	2 Capsules of 25 mg per week

*Adapted from Treatment Recommendations of a French Multidisciplinary Working Group by Peyron et al. (17).*

In case of a primary maternal *Toxoplasma* infection, to exclude fetal abnormalities, a monthly ultrasonographic monitoring is recommended until term. When amniocentesis is positive, ultrasounds must be checked every 2 weeks to monitor the brain anatomy of fetus. The main ultrasound findings associated with congenital toxoplasmosis are ventriculomegaly and intracranial calcifications. Prognosis of isolated fetal parenchymal cerebral lesions without ventriculomegaly were not related to neurological damage, instead hydrocephalus is associated with adverse neurological sequelae. The interaction between physicians and families is always important in managing a pregnancy complicated by fetal infection, termination of pregnancy is to be discouraged unless, in the opinion of experts, there is evidence of serious sequelae affecting the fetus (17, 63).

## Management of Neonates at Birth

All neonates at risk of congenital toxoplasmosis (proven maternal infection, with or without prenatal diagnosis) must perform a complete clinical and neurological check-up at birth, specific seroimmunological tests, direct and indirect dilated fundoscopy (to exclude chorioretinitis, or associated ophthalmological pathologies), and transfontanellar ultrasound examination (to rule out any ventricular dilatation, cerebral calcifications, porencephaly) (64).

Hepatic and cardiac ultrasound, brain computed tomography (CT) or magnetic resonance imaging (MRI) and electroencephalographic monitoring (EEG), are helpful when clinical and neurological symptoms are severe.

### Postnatal Diagnosis

**FIGURE 3:**
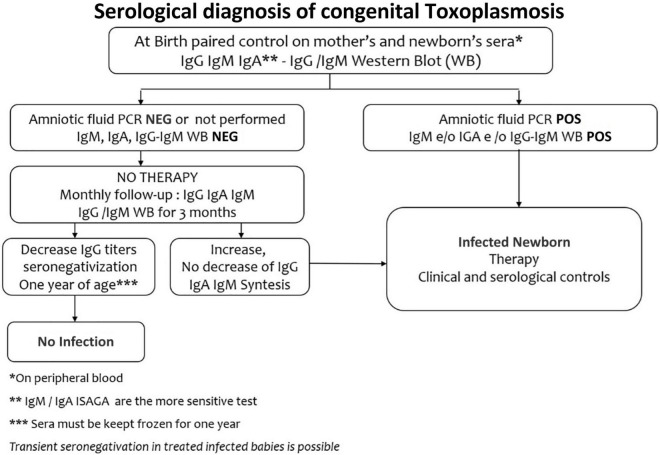
Serological screening of congenital toxoplasmosis.

IgM and IgA anti-Toxoplasma antibodies by Enzyme-Linked Immunosorbent Assay method (ELISA) or Immunosorbent Agglutination Assay (ISAGA) on a peripheral neonatal blood sample, represent the best markers of congenital infection, since these specific antibodies are produced by the neonate and cannot cross the placental barrier (Figure 3). With traditional tests for IgM and IgA antibodies, it is possible to diagnose at birth only 75% of infected newborns (6). IgM ISAGA is the more sensitive and specific test for the detection of Toxoplasma IgM (49). The absence of specific IgM and IgA antibodies does not exclude the infection since they may be not produced by congenitally infected infant in the first month of life (47). Furthermore, when maternal infection occurs in the late pregnancy, testing at birth may be falsely negative (17).

The IgG antibodies cross the placenta, so they are not a marker of congenital infection. Maternal anti-Toxoplasma IgG decline to disappear within 6–12 months. The persistence of IgG antibodies up to one year or their increase in the first months, lead to the diagnosis of congenital toxoplasmosis, whereas their negativity at one year, in subjects who did not receive any therapy, excludes the infection (65).

In newborns at risk of congenital toxoplasmosis, with positive IgG and negative IgM and IgA tests, a comparative mother-infant Western blot (WB) allows the early detection of synthesized neonatal antibodies, which have different antigenic specificity from maternal ones (66). Western blot has an excellent specificity (97–100%) and, in addition to traditional tests, will identify up to 96–98% of infected newborns. However, after the third month of life, the test becomes non-specific (67).

In infected infants, Interferon Ƴ release assay (IGRA) may be used to evidence lymphocytes activation and secretion of interferon Ƴ, following *in vitro* stimulation with *T. gondii* antigens (68).

If at birth there are insufficient data for the diagnosis, a definitive response can now be obtained much faster than in the past since confirmatory testing can be validated by reference laboratories within the first 60-90 days of life in almost all cases since Western blotting has been shown to establish diagnosis up to 3 months earlier than conventional serological methods (67, 69).

### Treatment of Infected Neonates

The efficacy of therapeutic protocols does not have the support of randomized controlled trials. Observational data describe that congenital toxoplasmosis has a good outcome and results in a normal neurological development when the treatment begins as soon as possible, both in pregnant mother and in newborn. Accordingly, delaying the therapy and/or neglecting subclinical infection increase the risk of serious disabilities (70, 71).

Synergistic effect of the combination therapy with pyrimethamine and sulfonamides against experimental toxoplasmosis, was observed in mice in the early 1950s. Studies performed decades ago provided the basis for the current recommendation for the combination of pyrimethamine with sulfadiazine or sulfadoxine and folinic acid as first-line treatment of toxoplasmosis in humans and, even today, it remains the gold standard.

Pyrimethamine and sulfonamide, because of their action on folate synthesis, act synergistically against *T. gondii*. Both drugs reduce the growth of the rapidly proliferating tachyzoites and prevent their transformation into new cysts, which are unsensitive to this treatment (72). Pyrimethamine is absorbed slowly but completely in the gastrointestinal tract. The serum half-life in the newborn is about 60 hours, and in cerebrospinal fluid reaches a concentration of about 10–20% of serum levels (17). Sulfadiazine seems to be the most active sulfonamide; its plasma half-life of 12–19 h makes it preferable to other sulfonamide and its concentration in the cerebrospinal fluid reaches 50% of the plasma concentration. It is excreted by the kidney, and its poor solubility can give crystalluria, which can be avoided with good patient hydration. The sulfadoxine, half-life of 120–195 h, allows for a simpler administration scheme (17).

There is no clear evidence about the comparative efficacy of the different postnatal treatment protocols, applied by different centers (14, 47, 60, 73). The French ones, brought together in a multidisciplinary team, have provided state-of-the-art care and an algorithm that optimizes outcomes for those suffering from this infection (17). Another protocol, adopted in several European countries, refers to the one published by Rima Mc Leod with a higher or a lower dose of pyrimethamine according to the severity of disease at birth (71).

Treatment should be continued for at least one year since a shorter therapy can lead to severe disabilities (60). Before starting the therapy, a G6PD deficiency must be ruled out, and throughout the whole treatment patients must be monitored clinically and serologically, to check the efficacy of therapy and the possible occurrence of side reactions (72).

Hematological adverse events may affect up to 30% of newborns. Use of antifolates and sulfonamides may result in a gradual bone marrow depression, more frequent in the first two months of life and mainly consistent in a reversible neutropenia. Folinic acid must always be associated for prevention and reduction of the hematological toxicities of the drugs. Sometimes anemia and thrombocytopenia are also present. The blood of patients must be therefore monitored, initially every 15 days, and later once a month. When neutrophils are <800/mm^3^, the therapy must be temporarily interrupted and resumed with the rising of white blood cells (17). No long-term hematological toxicity or late onset malignancies have been found. Gastrointestinal symptoms such as vomiting, diarrhea and lack of appetite are also occasionally described (74). A small percentage of studies (75) report dermatologic adverse events, including rash. Sulfonamide intolerance causes severe skin manifestations, such as Steven Johnson syndrome, and the treatment must be stopped immediately and permanently (17).

Azithromycin has good tissue and intracellular concentration and demonstrated *in vivo* activity against *T. gondii*. However, we have few data on its use in congenital infection. Again, there are no data in infants for therapy with Macrolides, Clindamycin, Atovaquone and immunotherapy (17, 60, 76).

Finally, we must obviously stress that breastfeeding and vaccination are strongly recommended.

## Ocular Toxoplasmosis

In congenital toxoplasmosis, chorioretinitis may be present even at birth, with chorioretinal scars or focal necrotizing retinitis. It may also appear unpredictably during childhood or adolescence, as a new manifestation or as a reactivation of a previous lesion (37). The symptoms are scotoma, pain, photophobia, blurred vision, and excessive watering of the eye (22).

A delay between maternal seroconversion and the beginning of treatment, and especially the presence of cerebral calcifications, are risk factors of retinochoroiditis during the first 2 years of life (77). In a prospective cohort study on 3 years old children with congenital toxoplasmosis, more than 90% of children with chorioretinitis had normal vision in the best eye, and only 9% had severe bilateral impairment (78).

In the active form, the specific therapy (Pyrimethamine + Sulfadiazine + folinic acid) must be applied for at least 1–2 weeks after resolution of clinical signs, the longest treatment being about 3 months. The efficacy of steroids in ocular toxoplasmosis has not been clearly demonstrated. Currently, they are used in association with the standard therapy only in severe inflammation, or when the lesions are close to the fovea or the optic disk. Corticosteroid therapy, without antiparasitic treatment, may result in large retinal lesions (60, 79).

A recent meta-analysis indicated that the combination of trimethoprim-sulfamethoxazole could be an alternative treatment (80). A long-term, intermittent regimen of this combination can be used in an effort to reduce the recurrence of chorioretinitis (81).

## Follow-Up

The follow-up of the newborn with congenital infection is mainly dedicated to the ophthalmological, neurological, auditory and serological aspects.

### Ophthalmological Follow-Up

Since the diagnosis of ocular toxoplasmosis remains fundamentally clinical, it is crucial to continue a long-term follow-up in all congenital patients. The risk of ocular affections persists throughout the whole life, even in treated children. In a follow-up with a median of 10.5 years, 29% of infected children born from treated mothers developed at least a new ocular lesion after the first one (82). It is therefore strongly recommended to plan a fundus check, performed by direct ophthalmoscopy, every three months in the first year of life, every six months in the second year and every further year, without age limits.

When checks are carefully performed and the pathology properly treated, the overall prognosis is satisfactory, and the consequences are rarely severe. The impact of retinochoroiditis and of associated eye pathologies neither reduce the visual performance of affected patients nor compromise the long-term quality of life (83).

### Neurological Follow-Up

A full evaluation of the neurobehavioral outcome of the newborn should be done in the first years of life. The predisposing factors for neurological anomalies are a lacked prenatal therapy and the presence at birth of chorioretinitis, possibly already accompanied by severe neurological signs, such as hydrocephalus, convulsions, muscle tone abnormalities. A transfontanellar ultrasound follow-up is recommended, looking for cerebral calcifications and ventricular dilatation.

Without cerebral lesions, there will be no neurological sequelae. In case of isolated calcifications, neurodevelopmental outcome is normal in most children and the calcifications may resolve during therapy (84). Indeed, the National Collaborative Chicago Based Congenital Toxoplasmosis reports that even in severe conditions, extremely rare at birth, a timely prenatal therapy, continued throughout the first year of life, led to a remarkable resolution of the neurological abnormalities (25).

As to the correlation between congenital toxoplasmosis and sensorineural deafness, data are very reassuring, since no infant treated for 12 months with an early specific therapy had sensorineural severe deficits. Accordingly, no association between *T. gondii* infection in pregnancy and hearing loss in offspring is recorded (85, 86). However, correlations between congenital toxoplasmosis and a high prevalence of hearing problems and language delays have been described in Brazil (Congenital Toxoplasmosis Brazilian Group) where the disease is more frequent and severe than in Europe (87).

The long-term impact of congenital toxoplasmosis on life quality and visual performance, was good in most of a cohort of adult individuals, who had been treated in pre-postnatal period. No limitations in cognitive functions were present, and the school level is not affected by this pathology (82).

### Serological Follow-Up

When diagnosis is not defined in the prenatal period (if performed) or at birth, and newborn is at risk of congenital infection, a serological follow-up must go on without starting any treatment that may mask the serology.

Serological test must be performed monthly in the first 3 months and after every 2 months, until one year of age. The absence of a congenital infection is defined by the negativization of specific antibodies within the first year of life, in absence of therapy. On the contrary, the increase of IgG or the appearance of IgM and/or IgA antibodies define the congenital infection (18).

When the maternal infection dates in the last weeks of pregnancy, the serology of the newborn at birth can be falsely negative. It is therefore recommended a monthly serological follow up without starting any treatment (18). A transient negativization of the IgG frequently happens during therapy for congenital infection. This however must not lead either to a treatment suspension or to question the diagnosis (17).

After the end of a full year of therapy, the IgG antibody titer may recover in 70–97% of cases. This antibody rebound is considered an antigenic re-expression that occurs when the drug pressure is over; if ophthalmological surveillance is warranted, prosecution of the treatment does not seem necessary (88, 89).

## Prevention of Primary *T. gondii* Infection in Pregnancy

The knowledge about *T. gondii* life cycle is crucial to reduce exposure to major risk factors, such as raw or undercooked meats (especially lamb and pork), poor hand hygiene and improper cleaning of cooking utensils, consumption of unfiltered water, gardening and other soil contact, exposure to cat litter and travels to high-incidence countries (90). Furthermore, the vaccination of food animals, the decontamination of all meats and products destined for undercooked consumption, and the vaccination of cats are ongoing intervention strategies to reduce *T. gondii* disease burden, across the general population.

However, special considerations must apply to all pregnant women, where health education approaches are mainly aimed at the prevention of congenital toxoplasmosis. Dietary recommendations and behavioral factors, including pet care and environmental measures, can help to reduce the risk of acquiring toxoplasmosis in pregnant women or women of childbearing age (91). Randomized controlled trials have shown little evidence that prenatal education has a positive effect in reducing seroconversion for toxoplasmosis in pregnancy (92). A recent systematic review on hygiene measures as primary prevention of toxoplasmosis during pregnancy suggests the efficacy of health education on *toxoplasma*-related knowledge, behavior, and risk of seroconversion in pregnancy (93).

**FIGURE 4:**
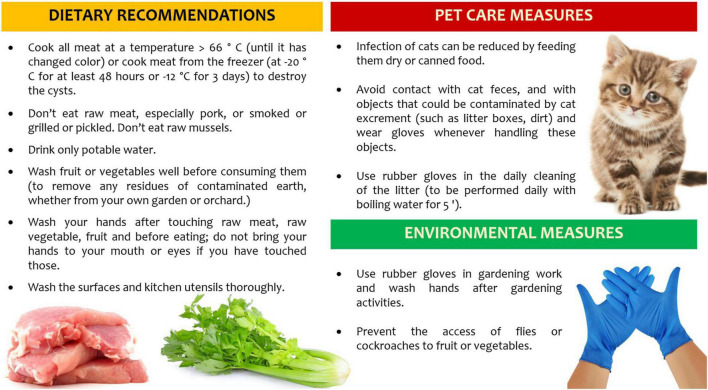
Recommendations to prevent *Toxoplasma* transmission during pregnancy.

Health care professionals must be aware of the risk factors and of the recommendations to give to seronegative pregnant women at the beginning of pregnancy (Figure 4): the knowledge of these preventive measures will have a positive impact.

## Conclusion

The correct hygienic-sanitary education of the seronegative pregnant woman (primary prevention), the systematic serological screening in pregnancy (secondary prevention) allowing early diagnosis and therapy of pregnant mother, the treatment of congenital infection and the follow-up of the newborn, have proved to be the cornerstones for addressing a public health problem such as congenital toxoplasmosis.

The development of new techniques for diagnosis of maternal infection, prenatal diagnosis and a therapeutic approach to limit vertical transmission and fetal injury of toxoplasmosis, can all concur for positive outcomes regarding health and quality of life.

Remarkable differences exist in public-health policies; research should be performed to assess the burden of congenital toxoplasmosis and the cost of care of the disease in each country (45). Furthermore, there is a need for implemented information programs and homogeneous guidelines, and more reference centers for the management of maternal or congenital infection, and for the post-natal follow-up of neonates with suspected congenital toxoplasmosis.

Despite great advances in basic clinical and scientific research, many questions remain to be addressed. We stress the necessity of less expensive serological tests, new drugs with less toxicity, more efficacy (allowing for a shorter treatment cycle), and able to eliminate resistant cysts (94, 95). Furthermore, improved pediatric formulations for established drugs would be useful (72).

Toxoplasmosis is still among the main infections that can be transmitted from mother to child and, when untreated, may have serious consequences; therefore, we must alert the health policy makers, not to make congenital toxoplasmosis a neglected disease.

## Author Contributions

LB and CT performed literature search and wrote the first draft of manuscript. CAu, CAc, FG, DD, and GS critically revised the manuscript. All authors approved the submitted and final versions of the manuscript.

## Conflict of Interest

The authors declare that the research was conducted in the absence of any commercial or financial relationships that could be construed as a potential conflict of interest.

## Publisher’s Note

All claims expressed in this article are solely those of the authors and do not necessarily represent those of their affiliated organizations, or those of the publisher, the editors and the reviewers. Any product that may be evaluated in this article, or claim that may be made by its manufacturer, is not guaranteed or endorsed by the publisher.

## Footnotes

1
https://pubmed.ncbi.nlm.nih.gov/
